# Higher Risk of Gastric *Helicobacter pylori* Infection in Patients with Periodontitis: A Nationwide Population-Based Retrospective Cohort Study in Taiwan

**DOI:** 10.3390/ijerph182111678

**Published:** 2021-11-07

**Authors:** Xin Li, Hitesh Singh Chaouhan, Ching-Hao Li, Tung-Min Yu, I-Kuan Wang, Cheng-Li Lin, Chi-Yuan Li, Kuo-Ting Sun

**Affiliations:** 1 Graduate Institute of Biomedical Sciences, China Medical University, Taichung 404, Taiwan; papa82988@gmail.com (X.L.); hschaouhan191986@gmail.com (H.S.C.); kevinli48@yahoo.com.tw (C.-H.L.); yu5523@gmail.com (T.-M.Y.); ikwang@seed.net.tw (I.-K.W.); 2 Division of Nephrology, Department of Internal Medicine, Taichung Veterans General Hospital, Taichung 404, Taiwan; 3 Division of Nephrology, China Medical University Hospital, Taichung 404, Taiwan; 4 Department of Internal Medicine, School of Medicine, China Medical University, Taichung 404, Taiwan; 5 Management Office for Health Data, China Medical University Hospital, Taichung 404, Taiwan; orangechengli@gmail.com; 6 Department of Anesthesiology, China Medical University Hospital, Taichung 404, Taiwan; 7 Department of Pediatric Dentistry, China Medical University Hospital, Taichung 404, Taiwan; 8 School of Dentistry, College of Dentistry, China Medical University, Taichung 404, Taiwan

**Keywords:** periodontitis, *Helicobacter pylori*, gastric infection, risk factors, cohort study

## Abstract

Periodontitis is the most prevalent chronic inflammatory oral disease that is characterized by tooth loss and is commonly associated with several systemic inflammatory diseases. Some epidemiological studies suggest that those suffering from periodontitis might be at a greater risk of developing gastric *Helicobacter pylori* (*Hp*) infection; however, evidence that showing the association between periodontitis and the risk of gastric *Hp* infection is less clear. We conducted a large-scale, population-based study in Taiwan with a 13-year follow-up period to evaluate the risk of gastric *Hp* in a periodontitis patient cohort. To conduct this study, we used epidemiological data from the Taiwanese Longitudinal National Health Insurance Research Database (NHIRD) from 2000 to 2013. We selected 134,474 participants (64,868 males and 69,606 females with a minimum age of 20 years), with and without periodontitis, and matched patient cohort groups for age, sex, index year, and co-morbidities. The Cox proportional hazards regression model was used to examine the risk of gastric *Hp* infection in patients with periodontitis. Patients with periodontitis exhibited a higher risk of developing gastric *Hp* infection compared to those individuals/groups without periodontitis (1.35 vs. 0.87 per 1000 person-years, adjusted the hazards ratio (aHR 1.52), and 95% confidence intervals (CIs) 1.38–1.67, *p* < 0.001). The risk of gastric *Hp* infection persisted even after stratifying by age (aHR = 1.96 (1.79–2.13) for 50–64 years and 1.70 (1.49–1.94) for ≥65 years), gender (aHR = 1.20 (1.11–1.29) for men), and presence of comorbidities of hypertension (aHR = 1.24 (1.11–1.38)), hyperlipidemia (aHR = 1.28 (1.14–1.42)), COPD (aHR = 1.45 (1.31–1.61)), CLD (aHR = 1.62 (1.47–1.77)) and CKD (aHR = 1.44 (1.04–1.99)). Overall, our findings showed that periodontitis patients have a greater risk for gastric *Hp* than individuals without periodontitis. Clinicians should perform regular good oral hygiene practices, along with newer treatments, for patients with periodontitis, especially those at higher risk of gastric *Hp* infection.

## 1. Introduction

Periodontitis is characterized as a chronic inflammatory oral disease that causes progressive loss of tooth-supporting tissues, such as gingiva, periodontal ligament, and surrounding alveolar bone, inevitably leading to complete tooth loss in older adults [1]. A four-year epidemiological study in the U.S. reported that 46% of adults aged 30 years were affected with periodontitis, and, among them, 8.9% had the worst periodontitis condition [2]. Moreover, a 17-year follow-up case-control study of periodontitis in Taiwan showed that the incidence of periodontitis steadily rose from 11.5% in 1997 to 19.59% in 2013 [3,4]. As a global disease burden, periodontitis is associated with various systemic inflammatory diseases, including atherosclerosis [5], cardiovascular diseases [6], respiratory disease [7], diabetes mellitus [8], rheumatoid arthritis [9], osteoporosis [10], dementia [11], and even gastric disease are linked with periodontitis [3,12]. Most researchers have speculated that these associations might be due to the induction of systemic inflammation, accompanied by a higher growth of pathogenic microorganisms in periodontal tissues that release inflammatory products into circulation.

*Helicobacter pylori* (*Hp*) is a microaerophilic, Gram-negative, and helical-shaped mobile bacterium [13]. It is one of the most common microbial infections in the human digestive tract, and is responsible for chronic gastritis, gastric and peptic ulcers, and gastric carcinoma [14,15]. An epidemiological survey found that approximately 50% of the world’s population is believed to have suffered from *Hp* infection, and the incidence rates of infection are generally increasing in developing countries, wherein the prevalence reported in adults is around 90% [16,17,18]. Earlier studies have indicated that the oral cavity is the primary reservoir of *Hp*, especially in periodontal plaques, dental pulps, the oral ulcerative area, and saliva, or where it serves as a possible route of transmission to other sites [19,20,21]. Oral *Hp*, generally linked to many oral diseases, e.g., periodontitis, might also be involved in gastric-related diseases because the presence of *Hp* in the oral cavity interferes with gastric *Hp* eradication therapy [22,23], and similar types of *Hp* strains from the oral cavity and the stomach have been isolated [24,25,26]. Concomitantly, the risks of incidence or recurrence of gastric *Hp* infection are often observed in individuals who harbor this organism in the oral cavity [27,28]. Avcu et al. [29] also found that stomach *Hp* infection could recur more frequently in patients with poor oral hygiene compared to patients with good oral hygiene. Therefore, these studies suggest that the oral cavity might serve as a potential reservoir of *Hp* before it is transmitted to the gastric mucosal area. Furthermore, they also observed oral–oral pathway as a primary transmission route in the promotion of extra-gastric *Hp* activity, where periodontal plaques, oral ulcerative areas, and saliva have been examined as vehicles for *Hp*, which may lead to recurrent gastric infections and the spread of infection to other sites.

*Hp* was first cultured from patients via gastric mucosal biopsy nearly 35 years ago [30]; the route of infection, reinfection, or oro-gastric transmission remains unclear and, thus, there are limited studies to describe the correlation between periodontitis and the risk of gastric *Hp* infection. Zhang et al. [31] surveyed clinical cases on a large scale in China and reported that the incidence rates of *Hp* infection and periodontal disease were 46.7% and 6.43%, respectively, in 54,036 cases. They also observed that severe periodontitis conditions (dental calculus and loose teeth) were associated with *Hp* infection and the relationship between oral *Hp* and gastric *Hp* infection. In another study, Zheng et al. [32] observed a higher *Hp* infection rate in both the mouths and stomachs of 70 elderly periodontitis patients and noted that this periodontitis condition in elderly people was possibly correlated with *Hp* stomach infection. However, the correlation between periodontitis and the risk of gastric *Hp* in the studies above was not well established because of discrepancies in the results, the fact that diagnosis was only confirmed through a review of medical records, and these studies were designed as cross-sectional or case control. To address these limitations, we employed the nationwide population database and designed a retrospective cohort study on a large scale, using the NHIRD medical claims data of Taiwanese people to investigate whether periodontitis increases the risk of developing gastric *Hp* infection.

## 2. Data and Methods

### 2.1. Data Source

In 1995, the Ministry of Health and Welfare of Taiwan established a single-payer National Health Insurance (NHI) program that provides universal and comprehensive health claims data for ~99% of Taiwanese people. The Longitudinal Health Insurance Database 2000 (LHID2000) is a subset of the National Health Insurance Research Database (NHIRD). NHIRD was set up and is managed by the National Health Research Institute of Taiwan. To conduct this study, we extracted data from LHID2000 which included 1 million individuals that were randomly selected from the NHI program. The database provides comprehensive de-identified healthcare information regarding demographic characteristics, including age, gender, date of birth and death, re-encoded identification numbers, number of inpatients and outpatients visiting, prescription drugs, medications, and diagnostic procedures from 2000 to 2013. In addition, all the diagnostic procedures were performed according to International Classification of Diseases, Ninth Revision, Clinical Modification (ICD-9-CM) guidelines and procedure codes. The current study was approved by the clinical review and research ethics committee of the China Medical University and Hospital (CMUH-104-REC2-115-R3).

### 2.2. Sample Participants

To examine the association between periodontitis and gastric *Hp* risk, we used the following two cohorts: one was a periodontitis cohort, and the other was a non-periodontitis cohort (comparison group). The periodontitis case group (*n* = 134,474, minimum age = 20 years) was comprised of newly diagnosed periodontitis patients (recognized from ICD-9-CM code 523.3 and 523.4) selected from 1 January 2000 to 31 December 2013. The date of the first diagnosis of periodontitis was characterized as the index date, and those periodontitis patients that were diagnosed with gastric HP were excluded before the index date. The comparison cohorts were matched at a 1:1 frequency with the case group by age (±5 year span), gender, and history of visits to a hospital within one year of the index date, and the same exclusion criteria were considered for this group. In addition, both the periodontitis cohort and the comparison cohort also excluded those who stopped using the health insurance before entry into the study or those who were younger than 20 years of age.

### 2.3. Study Outcome and Comorbidities

The primary outcome of this study was the occurrence of gastric *Hp* (ICD-9-CM code 041.86). All participants were followed-up with from the index date until *Hp* events occurred, they withdrew from the NHIRD program, death, or the end of study period (31 December 2013). We identified numerous comorbidities associated with gastric *Hp*, using the ICD-9 codes, before the index date and considered them as potential confounders. We considered the following comorbidities in this study: hypertension (ICD-9-CM code 401–405), diabetes (ICD-9-CM code 250), hyperlipidemia (ICD-9-CM code 272), chronic obstructive pulmonary disease (COPD, ICD-9-CM code 490–496), cirrhosis (ICD-9-CM code 571), and chronic kidney disease (CKD, ICD-9-CM code 585).

### 2.4. Statistical Analysis

The chi-squared test was used to evaluate the differences in the categorical variables, such as gender and comorbidities, while an independent two-tailed *t*-test was used for continuous variables, such as age, wherein mean age differences were analyzed between the two cohorts. The risk of gastric *Hp* in the periodontitis and non-periodontitis groups was determined using univariate and multivariate Cox-proportional hazards regression models, wherein the estimation and comparison were represented by hazards ratio (HRs), adjusted HRs, and a 95% confidence interval (CI). Moreover, after stratifying by age, gender, and the presence of comorbidities, the relative risk of gastric *Hp* between the cohorts (periodontitis vs. non-periodontitis) was estimated using the same hazards regression model. The incidence rates of gastric *Hp* risk were calculated by person-years. The cumulative incidence rate of gastric *Hp* risk was determined using the Kaplan–Meier model, and differences between groups were evaluated using the log-rank test. We used SAS software (version 9.4 for Windows; SAS Institute, Cary, NC, USA) and R software (R foundation for Statistical Computing, Vienna, Austria) to perform all the statistical analyses and the Kaplan–Meier model for all survival curve plots, respectively. Two-tailed *p*-values of <0.05 were considered to indicate statistical significance.

## 3. Results

**Figure 1:**
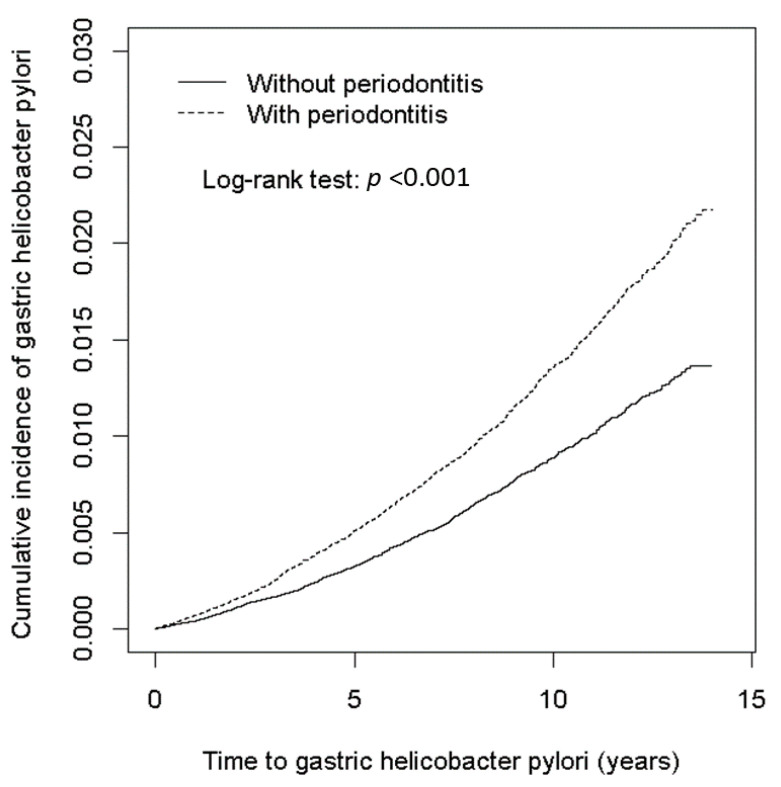
Cumulative risk of gastric *Hp* compared among in patients with and without periodontitis. Patients with periodontitis had a higher cumulative risk of gastric *Hp* than those without periodontitis.

In this study, we enrolled 134,474 participants (69,606 males and 64,868 females with a minimum age of 20 years), with and without periodontitis (Table 1). After using a chi-squared test, we observed that the distributions, stratified by age and sex between two groups, did not change, whereas the age distributions were different. The mean age in the study group was 43 years, and among them 48.2% were men. In the periodontitis group, there was a higher proportion of comorbidities, and hypertension, hyperlipidemia, COPD, and CLD were significant (*p* < 0.001) compared to the non-periodontitis group, except for diabetes mellitus and CKD. Figure 1 shows that the cumulative incidence rate of gastric *Hp* risk was significantly (*p* < 0.001) higher in the periodontitis cohort compared with the non-periodontitis cohort.

Table 2 shows the incidence rate, HRs, and aHRs for gastric *Hp* risk between the groups (periodontitis vs. non-periodontitis), where all study participants were stratified according to age, gender, and the presence of comorbidities. The incidence rates of developing gastric *Hp* in the patients with periodontitis and without periodontitis were 1.35 and 0.87, respectively. Compared with the comparison group, a significantly higher risk of gastric *Hp* (HRs = 1.53; 95% CI = 1.42–1.66) was observed in the periodontitis group, even after adjusting and stratifying sex, age, and comorbidities, while a similar periodontitis group showed 1.40 aHRs with 1.29–1.52 95% CI for gastric *Hp*. Moreover, also after adjusting and stratifying most factors according to gender, age, and comorbidities, we observed that patients with periodontitis that were 50–64 years old (aHR = 1.96; 95% CI = (1.79–2.13)) and ≥65 years (aHR = 1.70; 95% CI = (1.49–1.94), experienced hypertension (aHR = 1.24; 95% CI = (1.11–1.38)), hyperlipidemia (aHR = 1.28; 95% CI = (1.14–1.42)), COPD (aHR = 1.45; 95% CI = (1.31–1.61)), and CLD (aHR = 1.62; 95% CI = (1.47–1.77)), and exhibited a significantly (*p* > 0.001) higher gastric *Hp* risk.

Table 3 depicts the gastric *Hp* risk in the periodontitis and the non-periodontitis groups. Gastric *Hp* risk events were associated with age, gender, and comorbidities after adjusting and stratifying for other potential risk factors. As compared to non-periodontitis subjects, periodontitis patients were aged 20–49 years (aHRs = 1.40; 95% CI = (1.26–1.56)), 50–64 years (aHRs = 1.42; 95% CI = (1.23–1.64)) and ≥65 years (aHRs = 1.40; 95% CI = (1.09–1.81)); male patients (aHRs = 1.45; 95% CI = (1.30–1.63)) and female patients (aHRs = 1.35; 95% CI = (1.20–1.52)); and patients without comorbidities (aHRs = 1.57; 95% CI = (1.39–1.77) or with at least one comorbidity (aHRs = 1.33; 95% CI = (1.19–1.48) exhibited a significantly (*p* < 0.001) higher gastric HP risk.

Figure 1 shows the cumulative incidence rates of gastric *Hp* risk between the periodontitis and the non-periodontitis group via a Kaplan–Meier Cox regression model with a log-rank test. Patients with the periodontitis exhibited a significantly increased risk of gastric *Hp* events (Log-rank test; *p* < 0.001). From 1 January 2000 to the follow-up at the end of 31 December 2013, the incidence of gastric *Hp* risk events in the periodontitis group was significantly higher than in the corresponding comparison group.

## 4. Discussion

The current study is the first nationwide population-based retrospective cohort study to assess increased risk of gastric *Hp* in individuals with periodontitis in comparison to individuals without periodontitis. We found a significantly higher risk of gastric *Hp* in patients with periodontitis compared to those without periodontitis. Furthermore, the risk of gastric *Hp* was more likely to increase in older people, males, and those with or without any comorbidities. Even after adjusting for age, gender, and clinically selected comorbidities, the hazard of gastric *Hp* remained significant in periodontitis subjects compared to the comparison group. Thus, patients with periodontitis had a higher risk of gastric *Hp*.

It is important to consider that the incidence of gastric *Hp* significantly increases with age, in both the periodontitis and comparison groups; however, the crude and aHRs of the periodontitis vs. non-periodontitis group were higher in the youngest age group. Likewise, the incidence of gastric *Hp* increased with the presence of comorbidities in both the periodontitis and non-periodontitis cohorts. However, the crude and aHRs of the periodontitis vs. non-periodontitis group were higher in cases with no comorbidities of similar patient groups. This interesting observation reflects that periodontitis alone was correlated with the risk of gastric *Hp*; however, age and comorbidities may further modify this association (meaning more effects in the non- periodontitis group than in the periodontitis group).

As mentioned earlier, limited studies have focused on the relationship between periodontitis and the risk of gastric *Hp*, and most did not examine the precise correlation between periodontitis and gastric *Hp* infection risk [31,32]. In the current study, the association between periodontitis and a higher risk of gastric *Hp* infection was clearly examined. The higher prevalence of *Hp* in periodontitis patients may be linked to poor oral hygiene and the coexistence in both dental plaques and the stomach compared to individuals without periodontitis. Sambashivaiah et al. [33] reported that most patients with chronic periodontitis harbor a significantly higher *Hp* number than patients without periodontitis. According to observations of the different statuses of periodontitis, subjects with chronic periodontitis may have a greater risk of gastric *Hp* infection compared to mild-to-moderate periodontitis. Miyabayashi et al. [23] reported that a significantly higher prevalence of *Hp* in periodontal plaques might be an increasing risk factor for recurrent gastric infection. In addition, Song et al. [34] observed that gastric reinfection by *Hp* could happen in periodontitis subjects who received successful eradication therapy compared to those without therapy. Moreover, a recent clinical study on 698 *Hp*-infected gastric patients conducted in Taiwan showed that combinatorial treatments of periodontal therapy and gastric *Hp* eradication treatment significantly lowered the recurrence of gastric *Hp* infection, suggesting the oral–gastric transmission of *Hp* [35]. These findings conclude that developing new therapies and reducing periodontitis might decrease the risk of developing gastric *Hp*-related diseases. In contrast to these studies, one clinical-based study in Saudi Arabia, performed on 120 participants with good oral hygiene practices, showed that there was no significant association between gastric or oral *Hp* infection and periodontal disease [36]. In the present study, it is worth mentioning that periodontitis, with or without comorbidity, increases the risk of gastric *Hp* and, thereby, might be involved in the development and progression of many gastro-intestinal-related pathologies.

Several possible mechanisms have been suggested to explain how periodontitis occurs in oral cavity tissues, which may be linked to the risk of gastric *Hp* infection. First, an imbalance in oral microbiota due to periodontitis could induce systemic inflammation and aggravate the risk of gastric *Hp* infection/reinfection. Earlier studies have reported that the oral cavity is a gateway to the digestive tract, and could be a potential reservoir for *Hp* infection [23,29,37,38,39,40]; the presence of infection is generally linked to oral cavity damage and leads to an increase in gingival bleeding, alveolar bone resorption, and tooth loss during periodontitis. It was also found that oral–oral, fecal–oral, and oral–gastric routes are the most common routes for the spread of *Hp* infection at other sites. In earlier studies, individuals with periodontitis harbored a higher population of *Hp* at oral and gastric sites, which is a well-known cause of peptic ulcers [3,41]. The detection of *Hp* in the oral cavity tissues of gastritis patients using different methods raises the possibility of infection or reinfection in the stomach [42,43,44,45]. Furthermore, the failure of *Hp* treatment to eliminate *Hp* from the mouth could lead to recolonization in the stomach, thus accounting for the recurrence of gastric *Hp* infection and gastric-related diseases. Oral infection from periodontopathogenic bacteria may result in the spread of inflammation to the systemic circulation, leading to a chronic inflammatory state that promotes the risk of gastric *Hp* infection. Additionally, the greater invasive and proliferation rate of *Hp* in gastric mucosa epithelial cells could be involved in gastric-related disease induction, host immune response escape, and chronic inflammation. Contrary to the oral cavity, which presents as a potential reservoir for *Hp* bacterium, some authors believe that this bacterium does not consistently exist in the oral cavity environment but is transiently present at other sites because of the ingestion of contaminated foods and/or the uprising of bacteria from gastroesophageal reflux [45,46]. Other studies have also reported that the presence of periodontitis may facilitate the oral–gastric transmission of *Hp* and the colonization of this bacterium in the digestive tract [47,48,49]. Increased expression of CagA and IL-8 due to *Hp* infection in gastric tissues was previously found in chronic periodontitis patients [50]. Thus, periodontitis would facilitate a microenvironment for oro-gastric transmission of *Hp*, as well as stimulation of gastric epithelial mucosa cells to release cytokines under *Hp* infection. In addition, a higher number of *Hp* internalized in gastric epithelial cells could lead to damage to these cells, which allows *Hp* to invade the lamina propria and translocate to the gastric lymph nodes, which may promote chronically induced inflammation [51]. All these studies suggest that periodontitis may be a marker indicating proinflammatory or abnormal host immune responses that greatly predisposes the development of gastric *Hp* infection. However, the exact cause of gastric *Hp* risk in periodontitis patients remains unknown, though emerging evidence has suggested that inflammation may be one of the leading factors for developing a risk of gastric *Hp* infection. Consistently, we can also propose that inflammation may play an important role in the relationship between periodontitis and the risk of gastric *Hp* infection.

The present study has many strengths. First, it is a nationwide large population-based retrospective cohort study evaluating the relationship between periodontitis and gastric *Hp* infection risk. Second, the available clinical data on periodontitis were mainly based on NHIRD health claims data, reported by clinicians; thus, the study of periodontitis status in the subjects was more objective and precise when compared to self-analyzed data. Finally, using a retrospective cohort study design, the periodontitis status was examined before the risk of gastric *Hp* infection; thus, the possible association between periodontitis and gastric *Hp* risk could be more precisely investigated. However, the current study possesses some limitations. First, the NHIRD database did not provide detailed data related to patient body mass index, diet, lifestyle, living habits, oral hygiene practices, family history, and environmental factors, which are possible confounding factors. Second, health claims in the database do not collect important clinical variable data, including inflammatory markers, laboratory data, detailed dental reports, culture results, urinalysis records, and other pathologic reports. Third, periodontitis, gastric *Hp* risk and comorbidities were diagnosed using the ICD format, which depends on the specialist clinician’s performance. Checkups on a regular basis are performed to prevent negligence and misdiagnoses. Moreover, periodontitis had a severity classification, such as mild, moderate, and chronic [52]. Correlation among the stages of periodontitis and gastric *Hp* risk was not evaluated in this database.

## 5. Conclusions

Taken together, the present study shows that patients with periodontitis may be at greater risk of developing gastric *Hp* infection than individuals without periodontitis. The relationship between periodontitis and gastric *Hp* risk remained statistically significant, even after adjusting for age, sex, and presence of comorbidities. As periodontitis is associated with several systemic inflammatory diseases, including gastric diseases, further studies would be beneficial to suggest that medical professionals perform regular good oral hygiene, along with newer treatments for periodontitis patients that have a higher risk of extra-gastro reservoir *Hp* infection.

## Figures and Tables

**Table 1:** Baseline characteristics for individuals with and without periodontitis.

Variables	Periodontitis				*p*-Value ^#^
	Yes		No		
	N = 134,474		N = 134,474		
	n	%	n	%	
Age					0.99
20−49	91,982	68.4	91,982	68.4	
50−64	29,720	22.1	29,720	22.1	
≥65	12,772	9.50	12,772	9.50	
Mean (±SD)	42.8	15.5	43.1	15.1	<0.001
Gender					0.99
Women	69,606	51.8	69,606	51.8	
Men	64,868	48.2	64,868	48.2	
Comorbidity					
Hypertension	24,003	17.9	26,867	20.0	<0.001
Diabetes mellitus	4451	3.31	4459	3.32	0.93
Hyperlipidemia	16,207	12.1	23,450	17.4	<0.001
COPD	17,984	13.4	23,225	17.3	<0.001
CLD	20,582	15.3	28,884	21.5	<0.001
CKD	1206	0.90	1244	0.93	0.44

CKD, chronic kidney disease; CLD, chronic liver disease and cirrhosis; COPD, chronic obstructive pulmonary disease; SD, standard deviation. ^#^ chi-squired test and independent two-tailed *t*-test.

**Table 2:** The event numbers, incidence rates, and hazard ratios (HRs) of gastric HP for potential risk factors.

	Event	PY	Rate ^#^	Crude HR (95% CI)	Adjusted HR ^†^ (95% CI)
Periodontitis					
No	955	1,095,771	0.87	1.00	1.00
Yes	1565	1,157,725	1.35	1.53 (1.42, 1.66) ***	1.40(1.29, 1.52) ***
Age					
20–49	1439	1,595,060	0.90	1.00	1.00
50−64	816	475,470	1.72	1.96 (1.79, 2.13) ***	1.52 (1.38, 1.67) ***
≥65	265	182,966	1.45	1.70 (1.49, 1.94) ***	1.10 (0.95, 1.28)
Gender					
Women	1207	1,177,880	1.02	1.00	1.00
Men	1313	1,075,616	1.22	1.20 (1.11, 1.29) ***	1.19 (1.10, 1.28) ***
Comorbidity					
Hypertension					
No	1816	1,866,367	0.97	1.00	1.00
Yes	704	387,128	1.82	1.94 (1.78, 2.12) ***	1.24 (1.11, 1.38) ***
Diabetes mellitus					
No	2409	2,190,127	1.10	1.00	1.00
Yes	111	63,369	1.75	1.67 (1.38, 2.02) ***	0.91 (0.75, 1.11)
Hyperlipidemia					
No	1914	1,950,907	0.98	1.00	1.00
Yes	606	302,588	2.00	2.12 (1.94, 2.33) ***	1.28 (1.14, 1.42) ***
COPD					
No	1953	1,948,333	1.00	1.00	1.00
Yes	567	305,163	1.86	1.96 (1.78, 2.15) ***	1.45 (1.31, 1.61) ***
CLD					
No	1733	1,856,621	0.93	1.00	1.00
Yes	787	396,874	1.98	2.17 (1.99, 2.36) ***	1.62 (1.47, 1.77) ***
CKD					
No	2482	2,238,429	1.11	1.00	1.00
Yes	38	15,066	2.52	2.48 (1.80, 3.42) ***	1.44 (1.04, 1.99) *

CI, confidence interval. CKD, chronic kidney disease; CLD, chronic liver disease and cirrhosis; COPD, chronic obstructive pulmonary disease. HR, hazard ratio; PY, person-years; ^#^ Incidence rate per 1000 person-years; ^†^ Multivariable analysis including age, gender, and comorbidities of hypertension, diabetes mellitus, hyperlipidemia, COPD, CLD, and CKD. * *p* < 0.05, *** *p* < 0.001.

**Table 3:** Incidences and hazard ratios of gastric *Helicobacter pylori* for individuals with and without periodontitis.

Variables	Periodontitis						Crude HR (95% CI) Adjusted HR ^†^	Crude HR (95% CI) Adjusted HR ^†^
	No			Yes				
	Event	PY	Rate ^#^	Event	PY	Rate ^#^		
Age								
20−49	555	781,062	0.71	884	813,998	1.09	1.51 (1.36,1.68) ***	1.40 (1.26, 1.56) ***
50−64	307	231,219	1.33	509	244,251	2.08	1.55 (1.35, 1.79) ***	1.42 (1.23, 1.64) ***
≥65	93	83,490	1.11	172	99,476	1.73	1.53 (1.19, 1.97) **	1.40 (1.09, 1.81) **
Gender								
Women	478	576,370	0.83	729	601,510	1.21	1.45 (1.29, 1.63) ***	1.35 (1.20, 1.52) ***
Men	477	519,401	0.92	836	556,215	1.50	1.61 (1.44, 1.81) ***	1.45 (1.30, 1.63) ***
Comorbidity ^§^								
No	446	736,978	0.61	628	670,052	0.94	1.53 (1.35, 1.72) ***	1.57 (1.39, 1.77) ***
Yes	509	358,793	1.42	937	487,673	1.92	1.33 (1.19, 1.48) ***	1.33 (1.19, 1.48) ***

CI, confidence interval; HR, hazard ratio; PY, person-years; ^#^ Incidence rate per 1000 person-years. ^†^ Multivariable analysis including age, gender, and comorbidities of hypertension, diabetes mellitus, hyperlipidemia, COPD, chronic liver disease (CLD), and chronic kidney disease (CKD). ^§^ Individuals with any comorbidity of hypertension, diabetes mellitus, hyperlipidemia, COPD, CLD, and CKD were classified into the comorbidity group. ** *p* < 0.01, *** *p* < 0.001.

## Data Availability

Original data will be available upon request.
